# Low-Dose Prospectively Electrocardiogram-Gated Axial Dual-Source CT Angiography in Patients with Pulsatile Bilateral Bidirectional Glenn Shunt: An Alternative Noninvasive Method for Postoperative Morphological Estimation

**DOI:** 10.1371/journal.pone.0094425

**Published:** 2014-04-15

**Authors:** Xiaopeng Ji, Bin Zhao, Zhaoping Cheng, Biao Si, Zhiheng Wang, Yanhua Duan, Pei Nie, Haiou Li, Shifeng Yang, Hui Jiao, Ximing Wang

**Affiliations:** 1 Shandong Medical Imaging Research Institute, Shandong University, Jinan, People's Republic of China; 2 Cardiovascular Institute of Jinan Military district, Jinan, People's Republic of China; University of Groningen, Netherlands

## Abstract

**Objective:**

To explore the clinical value of low-dose prospectively electrocardiogram-gated axial dual-source CT angiography (low-dose PGA scanning, CTA) in patients with pulsatile bilateral bidirectional Glenn shunt (bBDG) as an alternative noninvasive method for postoperative morphological estimation.

**Methods:**

Twenty patients with pulsatile bBDG (mean age 4.2±1.6 years) underwent both low-dose PGA scanning and conventional cardiac angiography (CCA) for the morphological changes. The morphological evaluation included the anatomy of superior vena cava (SVC) and pulmonary artery (PA), the anastomotic location, thrombosis, aorto-pulmonary collateral circulation, pulmonary arteriovenous malformations, etc. Objective and subjective image quality was assessed. Bland–Altman analysis and linear regression analyses were used to evaluate the correlation on measurements between CTA and CCA. Effective radiation dose of both modalities was calculated.

**Results:**

The CT attenuation value of bilateral SVC and PA was higher than 300 HU. The average subjective image quality score was 4.05±0.69. The morphology of bilateral SVC and PA was displayed completely and intuitively by CTA images. There were 24 SVC above PA and 15 SVC beside PA. Thrombosis was found in 1 patient. Collateral vessels were detected in 13 patients. No pulmonary arteriovenous malformation was found in our study. A strong correlation (R^2^>0.8, *P*<0.001) was observed between the measurements on CTA images and on CCA images. Bland–Altman analysis demonstrated a systematic overestimation of the measurements by CTA (the mean value of bias>0).The mean effective dose of CTA and CCA was 0.50±0.17 mSv and 4.85±1.34 mSv respectively.

**Conclusion:**

CT angiography with a low-dose PGA scanning is an accurate and reliable noninvasive examination in the assessment of morphological changes in patients with pulsatile bBDG.

## Introduction

**Figure 1:**
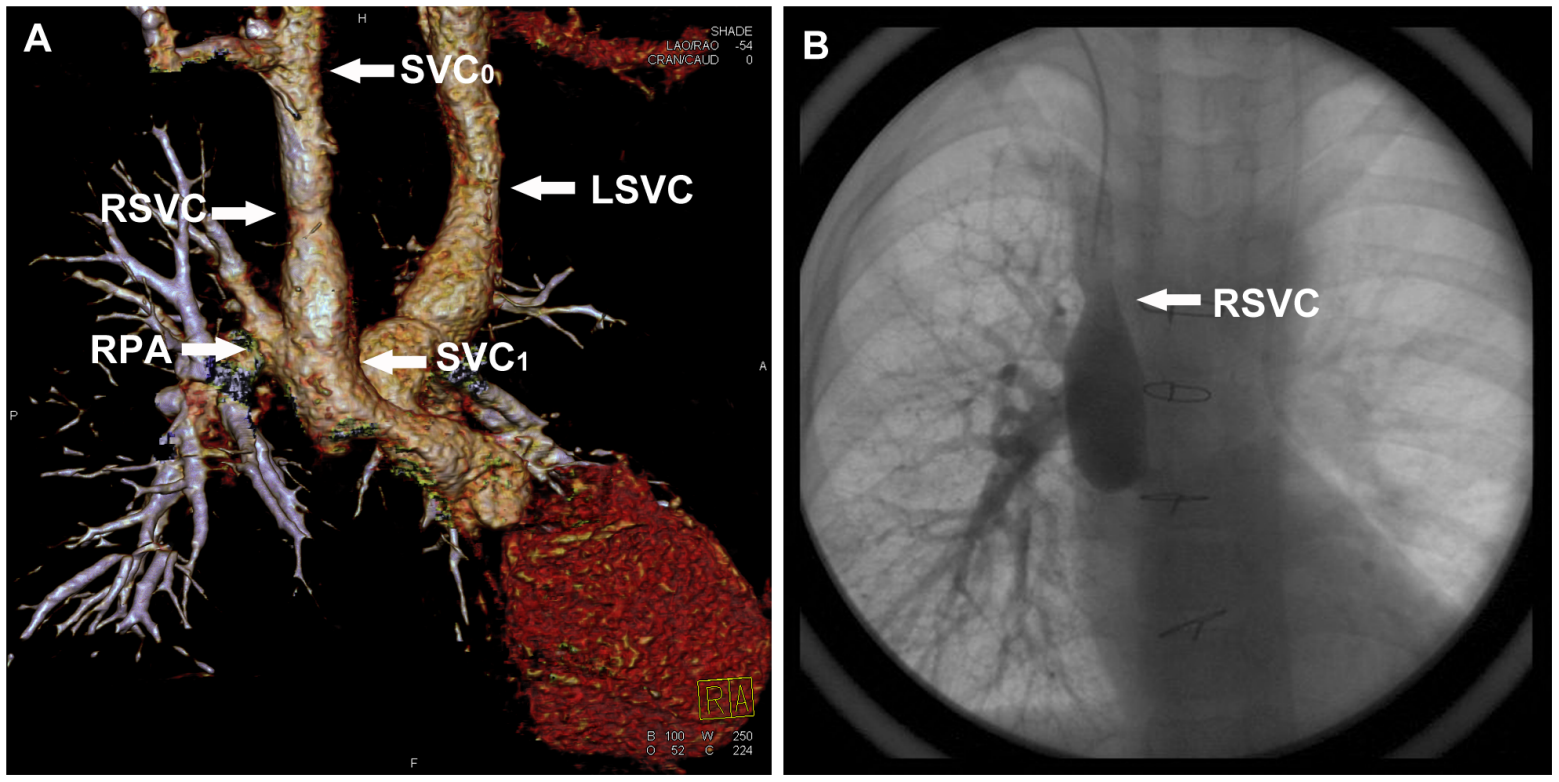
A 6-year-old boy underwent low-dose PGA scanning with 80 kV and 140 mAs/rot (effective dose, 0.77 mSv). **A**. Volume-rendered image (right lateral view) shows the morphologic measurements of SVC and the dilation of the proximal part of RSVC. **B**. CCA image confirms the dilation. *RSVC* right superior vena cava, *RPA* right pulmonary artery, *LSVC* left superior vena cava. SVC_1_ the proximal end of SVC, SVC_0_ the distal end of SVC.

Historically the Bidirectional Glenn shunt (BDG) was a palliative surgical procedure typically done in children with a single effective ventricle as an intermediate step before completion of a Fontan operation. The bilateral BDG (bBDG) is an end-to-side anastomosis between the bilateral superior vena cava (SVC) and the pulmonary artery (PA) respectively (Fig. 1A). Compared with the standard unilateral BDG, the bBDG has been found to be associated with a higher operative mortality, an increased risk of thrombus formation, and a lower conversion rate to the “Fontan-type” circulation [1], [2]. The role and effect of pulsatile pulmonary blood flow at the time of BDG is controversial. Several studies show a modest increase in arterial oxygen saturations and pulmonary artery growth for patients with anterograde pulmonary blood flow after BDG procedure [3], [4]. However, Ferns SJ, et al found that a higher postoperative complication rate was associated with pulsatile pulmonary blood flow [5]. Therefore, the postoperative estimation of bBDG procedure is extremely important.

There is no doubt that medical imaging plays a critical role in the postoperative estimation. Conventional cardiac angiography (CCA), which is routinely performed after bBDG, gives an accurate estimation of the morphological and hemodynamic information. However, with the improvement in noninvasive imaging modalities, as well as the technical difficulty, expense, and ionizing radiation exposure (for instance, 4.6 mSv for diagnostic catheterization and 6.0 mSv for therapeutic catheterization [6]–[9]) of CCA, there has been increasing interest in noninvasive evaluation. Echocardiography, the primary noninvasive imaging modality, is often limited by poor acoustic windows due to a large body habitus or immediate postoperative status. Cardiovascular magnetic resonance (CMR) which has been verified to provide anatomic and functional information without ionizing radiation, has been incorporated into the postoperative evaluation of patients with bBDG [10]. However, CMR is resource-intensive, technically challenging, and requires general anaesthesia or deep sedation for most children to avoid movement and imaging distortion [10]–[12]. Anesthetics by gas or by continuous infusion must be maintained throughout the MRI study [13]. Therefore, the operational risks of general anesthesia or deep sedation must be considered for these patients during the long acquisition time of CMR [14].

Recently, multidetector computed tomography angiography (CTA) with high spatial resolution and reduced acquisition time has been validated as a precise examination in the evaluation of children with repaired congenital heart diseases (CHD), and only short time sedation (chloral hydrate) rather than general anesthesia was used in uncooperative patients [15]–[18]. Prospectively electrocardiogram-gated axial (PGA) technique has been widely used in children suffering from CHD with a low radiation dosage of 0.26–0.38 mSv in recent years [19]–[21]. To the best of our knowledge, no study published study has evaluated the low-dose PGA protocol in patients after pulsatile bBDG procedure. The goal of this prospective trial of low-dose prospectively electrocardiogram-gated axial dual-source CT angiography (low-dose PGA scanning, CTA) versus CCA in children after pulsatile bBDG procedure was to test the hypothesis that CTA can provide accurate morphological information with low radiation dose.

## Materials and Methods

### 2.1 Ethics Statement

This study received approval from the Shandong Medical Imaging Research Institute ethics board. The possible adverse effects of contrast medium injection and radiation exposure as well as the complications of CCA were explained to all patients (the guardians of patients). Written informed consent was obtained from all patients (the guardians of patients) to prospectively enter this research.

### 2.2 Patients

Twenty children underwent pulsatile bBDG at a single institution. Both low-dose PGA scanning and CCA were performed in all patients (mean age 4.2±1.6 years, range 2–6 years; 15 males and 5 females; mean body weight 15.4±3.7 kg, range 10–21 kg) as a routine during a follow-up period of 6 to 12 months between April 2011 and July 2012.

### 2.3 Low-dose PGA scanning protocol

All examinations were performed on a first generation dual-source CT scanner (Siemens Somatom Definition, Siemens Healthcare, Forchheim, Germany). Younger children (≤15 kg) were sedated with oral chloral hydrate (50 mg/kg) [22] under the supervision of a pediatrician. Anesthesia was not given. Older children (>15 kg) usually responded well to verbal reassurance, and were usually relatively cooperative and good at breath holding. The scanning was performed in caudo-cranal direction from the bottom of the heart to the thoracic inlet.

CT parameters were as follows: 0.33 s gantry rotation time, 2×32×0.6 mm detector collimation, a slice collimation 2×64×0.6 mm by z-flying focal spot technique, 80 kV tube voltage and weight adapted setting for tube current (10 mAs/rot per kg up to 6 kg then 5 mAs/rot per kg up to 140 mAs/rot). We did not use β-blocker therapy to decrease the heart rate. The data acquisition window was set at 40% of the R-R interval.

Iodinated contrast medium (Schering Ultravist, Iopromide, 350 mg I/ml, Berlin, Germany) was injected via unilateral peripheral veins in the elbow or back of the hand at a volume of 2.0 ml/kg body weight with a saline chaser of 1.0 ml/kg body weight [19]. The delay between the start of injection and the start of data acquisition was set at 40 seconds. Injection rate was calculated at total injected volume divided by 30. For example, a 15 kg child with peripheral access would be injected with 30 ml of contrast medium and 15 ml saline at 1.5 ml/s.

### 2.4 CT data post-processing

Images were reconstructed with a slice thickness of 0.75 mm and increment of 0.5 mm using a medium smooth-tissue convolution kernel (B26f). All images were anonymous and transferred to an external workstation (Multiple Modality Workplace, Siemens Healthcare, Forchheim, Germany) for further analysis. In addition to the CT axial slices, Multiplanar reformation (MPR), maximum intensity projection (MIP) and volume rendering (VR) were used for image interpretation.

### 2.5 Assessment of image quality of CTA

As the postoperative changes of bilateral SVC and PA drawed pediatric cardiologists' attention, we paid great attention to the morphological evaluation of SVC and PA. Intraluminal attenuation and image noise of bilateral SVC and PA were assessed as objective image quality parameters. The magnitude and uniformity of arterial enhancement was measured using 1.0 cm^2^ circular ROIs at different regions. The noise was defined as the standard deviation of the attenuation value.

Blinded to the results of CCA findings, two cardiac radiologists with more than 5 years' experience respectively interpreted the subjective image quality of great vessels (mainly bilateral SVC and PA) using a 5-grade scoring system (5, excellent; 4, good; 3, fair; 2, insufficient for complete evaluation; 1, not interpretable) [21]. Grades 3, 4 and 5 were considered sufficient for complete diagnosis. For any disagreement between the two observers, consensus agreement was achieved.

### 2.6 Postoperative morphological evaluation of CTA

#### 2.6.1 Morphological measurements of SVC and PA

The diameters of the proximal (SVC_1_) and distal (SVC_0_) part of SVC, as well as the maximum diameter (SVC_max_) were measured (Fig. 1A). As the proximal end of SVC was stitched with PA and impacted more obviously by blood flow, SVC_0_ was chosen as the original diameter of SVC and the comparison between SVC_max_ and SVC_0_ was as follows: (SVC_max_ - SVC_0_)/SVC_0_×100%. The dilation of SVC was observed visually according to VR image.

The diameters of the prebranching point (PA_0_) of PA and the descending aorta at the diaphragm (DA) were measured, and McGoon ratio =  (LPA_0_+ RPA_0_)/DA. The stenosis of PA was assessed visually according to VR image.

The measurements were carried out three times in the MPR images and the average value was adopted. If the section of measurement was not circular, the caliber was quantified using 2 perpendicular measurements of the diameter on the transverse section. The represented diameter was the root of the multiple of these 2 perpendicular measurements.

#### 2.6.2 The anastomotic relationship between SVC and PA

**Figure 2:**
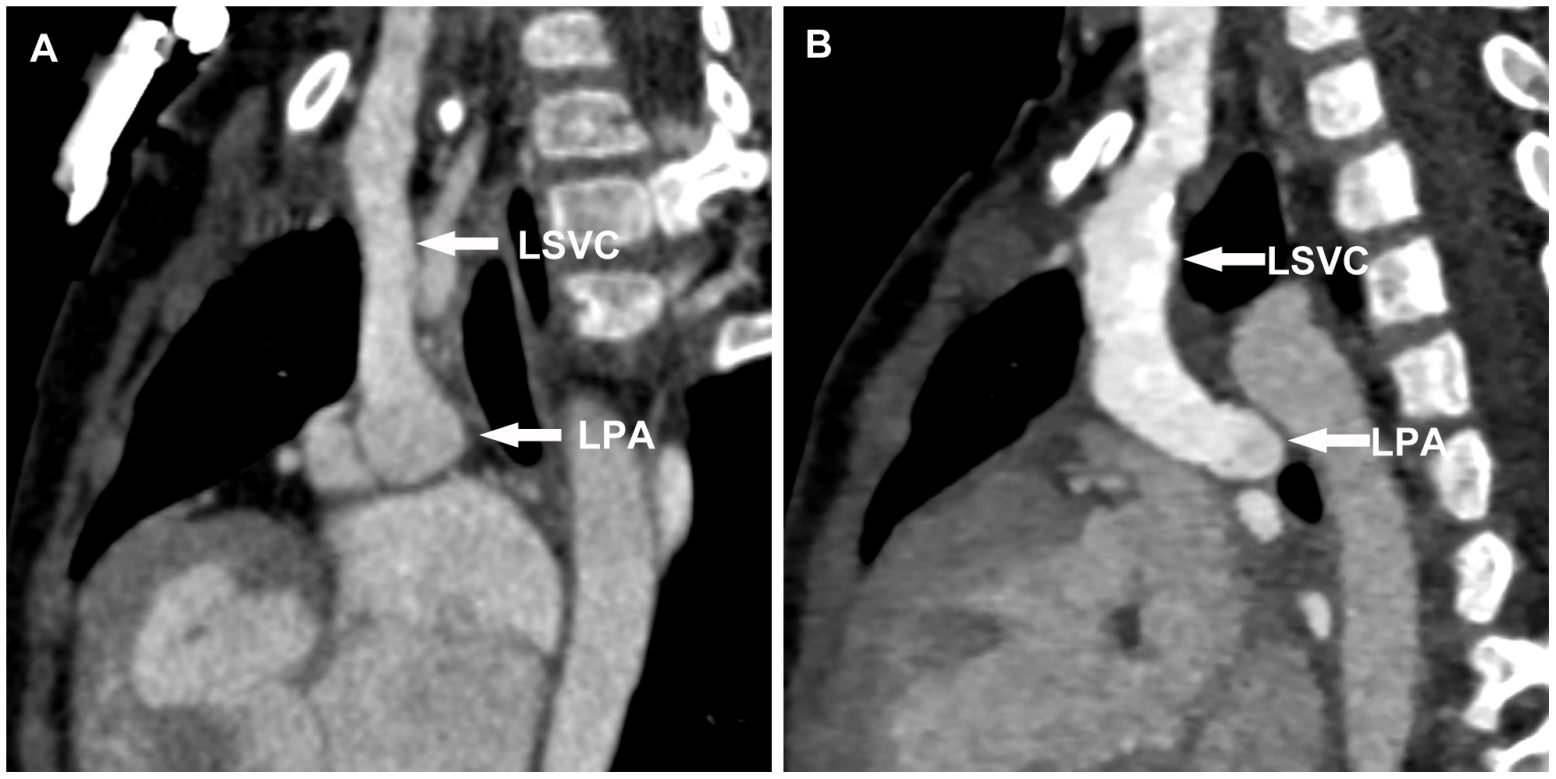
The anastomotic location between SVC and PA. **A**. Multiplanar reformation image shows that SVC was above PA when it located at ten to two o'clock of PA on the sagittal section. **B**. Multiplanar reformation image shows that SVC was beside PA at six to twelve o'clock of PA on the sagittal section. *LSVC* left superior vena cava, *LPA* left pulmonary artery.

The anastomotic location between SVC and PA was observed. We defined that SVC was above PA when it located at ten to two o'clock of PA on the sagittal section (Fig. 2A), while SVC was beside PA at six to twelve o'clock of PA (Fig. 2B).

#### 2.6.3 Thrombosis, aorto-pulmonary collateral circulation and pulmonary arteriovenous malformations

We paid great attention to thrombosis which is one of the severe postoperative complications of this procedure [23], [24]. An aorto-pulmonary collateral vessel (APC) was defined as one arising from the arterial circulation that had a discretely identifiable origin and perfused the pulmonary parenchyma [25]. APCs may form in patients postoperatively as a result of decreased pulmonary blood flow or pulmonary vein stenosis [26], and they were quantified by three indexes: the presence or absence of collateral vessels, the number of collateral vessels identified and the diameter of the proximal end of the most obvious collateral vessel (APC_max_). Another potential complication is the development of pulmonary arteriovenous malformations (AVMs). Pulmonary AVMs are postulated to occur because the SVC blood lacks a humoral factor from the liver which is needed to prevent the development of AVMs in the lungs [27]. Therefore, whether pulmonary AVMs existed also interested us.

### 2.7 Conventional cardiac angiography

CCA was performed by means of a Phillips' machine (Allura CV20, Phillips Medical Systems, The Netherlands) using a 4-6 Fr catheter 2 or 3 days after low-dose PGA scanning in all patients. They were kept fasting for 3–4 hr prior to the procedure. The procedure was done under general anesthesia with Midazolam (0.1 mg/kg) and Ketamine (1–2 mg/kg). Groin was cleaned and local anesthesia was given. All children were restrained by tying them over the board so as to avoid unnecessary movements. Arterial and venous access was obtained after puncturing right or left femoral artery and vein as well as unilateral or bilateral internal jugular veins by Seldinger's technique. Heparin was given 80 units/kg at the beginning of the procedure. Angiographic pictures were recorded on the Phillips' machine. The contrast medium used for angiography was non-ionic (Schering Ultravist, Iopromide, 350 mg I/ml, Berlin, Germany) with total dose limited to 5 ml/kg, and the average amount of contrast enhancement was 4.6±0.3 ml/kg.

As for the morphological assessment, biplane anteroposterior and lateral projection aortography by internal jugular vein was performed to evaluate the anatomy of SVC and PA, the anastomotic location between SVC and PA as well as thrombosis and pulmonary AVMs. Anteroposterior, lateral and left anterior oblique projection aortography or selective angiography was performed to observe the amount of APCs and APC_max_. With regard to the hemodynamic evaluation, the oxygen saturation of cardiac atrium, ventricle, the aorta and PA as well as the pressures of cardiac ventricle, aorta, main pulmonary artery, bilateral SVC and PA were measured. Since we focused on the morphological evaluation, the corresponding measurements of CCA were recorded and compared with those of low-dose PGA scanning while the hemodynamic data of CCA was not listed below.

### 2.8 Radiation dose estimations

The volume CT dose index and dose length product (DLP) were obtained from the information generated by the CT system and the effective radiation dose was calculated from the DLP as follows. The recorded DLP was multiplied by 2.3 to adapt it to the 16-cm phantom, because on the machine used for the study, the DLP for body surface area was given on a 32-cm phantom and 2.3 is scanner specific for pediatric examinations at 80 kV as provided by the manufacturer. The corrected DLP value was then multiplied by the infant-specific conversion coefficient given for a 16-cm phantom: 0.018 mSv/(mGy•cm) between 1 year and 6 years of age [28].

As for CCA, the dose-area product was displayed by the fluoroscopy system itself and the infant-specific coefficient 0.41 mSv/(Gy•cm^2^) [29] was used to convert the dose-area product into the effective dose.

### 2.9 Statistics

Statistical analysis was performed by SPSS 17.0 software (SPSS, Chicago, IL, USA) and Microsoft Windows Excel 2007 (Microsoft Co., Redmond, Washington, USA). Results were expressed as means ± standard deviations for quantitative variables and as frequencies or percentages for categorical variables. Interobserver agreement on grades of subjective image quality was assessed by kappa statistics (*κ*>0.81, excellent agreement; *κ* = 0.61–0.80, good agreement). With CCA results as the standard, linear regression analysis and Bland–Altman analysis were used to evaluate the correlation on measurements between CTA and CCA, and Pearson's correlation coefficient was calculated (R^2^>0.8, strong correlation). *P*<0.05 was considered statistically significant.

## Results

The CTA data of twenty children with pulsatile bBDG were successfully acquired. All patients were in sinus rhythm at the time of data acquisition. Mean heart rate was 98±11.7 bpm (range 77–119), mean heart-rate variability was 10.8±5.8 bpm (4–23). Data acquisition took place at every 2^nd^ or 3^rd^ heart beat (2^nd^: n = 15, 3^rd^: n = 3, 2^nd^ and 3^rd^: n = 2). Mean scan length was 109.7±15.1 mm (85–136), resulting in a mean of 6±1 (5–8) slab acquisitions per examination. Mean scan time was 6.6±1.1 s (4.79–8.63).

### 3.1. Image quality of CTA

Objective image quality parameters are listed in Table 1. All examinations showed diagnostic image quality, with no examination rated non-diagnostic (5, n = 5; 4, n = 11; 3, n = 4). The average subjective image quality score was 4.05±0.69, and 4.15±0.67 for reader 1 and 4.00±0.73 for reader 2 respectively. Overall interobserver agreement on subjective image quality was good (*κ* = 0.76, *P*<0.05). Artifacts due to respiratory motion were present in 6 examinations without impairing diagnostic quality.

**Table 1:** Objective image quality parameters.

	Attenuation(HU)	Image noise(HU)
**RSVC**	353.8±60.4	24.5±5.4
LSVC^*^	351.1±52.3	26.4±4.1
**RPA**	356.5±67.6	24.5±4.4
**LPA**	345.7±57.0	25.2±3.7

Notes: *RSVC* right superior vena cava, *LSVC* left superior vena cava, *RPA* right pulmonary artery, *LPA* left pulmonary artery. *LSVC was filled with thrombus in one patient, so LSVC was evaluated in nineteen patients.

### 3.2 Postoperative morphological evaluation

#### 3.2.1 Morphological measurements of SVC and PA

**Figure 3:**
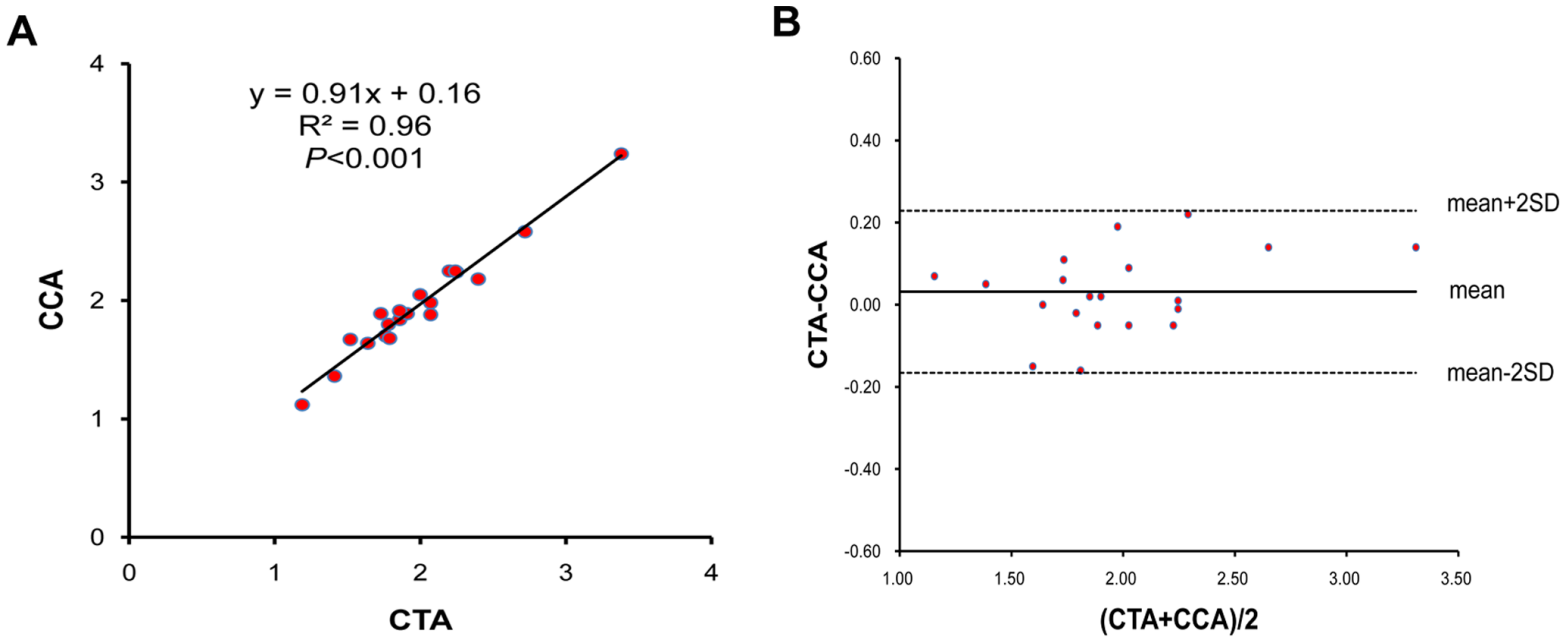
Correlation of McGoon ratio obtained by CTA and CCA. **A**. Two modalities showed a strong correlation. **B**. Bland–Altman plots illustrated bias and limits of agreement of McGoon ratio using CTA when compared with CCA.

The measurements were listed in Table 2. A strong correlation (R^2^>0.8, *P*<0.001) was observed between the measurements on CTA images and on CCA images. Bland–Altman analysis demonstrated a systematic overestimation of the measurements by CTA (the mean value of bias>0) [14]. The McGoon ratio obtained by CTA and CCA was 1.99±0.48 mm (1.19–3.38) and 1.96±0.45 mm (1.12–3.24) respectively, and there was a strong correlation (R^2^ = 0.96, *P*<0.001) (Fig. 3A). Bland–Altman analysis showed a systematic overestimation of the measurements by CTA (bias 0.032±0.10 mm) (Fig. 3B).

**Table 2:** Morphological measurements of CTA and CCA.

	the diameters* (mm)		comparison between CTA and CCA	
	CTA	CCA	linear regression analyses(R^2^)	Bland–Altman analysis(bias)(mm)
RSVC_0_	10.5(9.6 to 11.3)	10.4(9.6 to 11.3)	0.89	0.11±0.60
RSVC_max_	13.2(11.8 to 14.6)	13.0(11.6 to 14.4)	0.96	0.15±0.64
RSVC_1_	12.5(11.2 to 13.7)	12.3(11.1 to 13.6)	0.95	0.14±0.63
LSVC_0_**	9.4(8.6 to 10.1)	9.3(8.6 to 10.0)	0.89	0.12±0.52
LSVC_max_**	10.5(9.5 to 11.5)	10.3(9.4 to 11.3)	0.92	0.16±0.57
LSVC_1_**	10.1(9.1 to 11.2)	10.0(9.0 to 11.0)	0.94	0.13±0.53
RPA_0_	10.6(9.5 to 11.7)	10.5(9.4 to 11.7)	0.95	0.11±0.53
LPA_0_	9.2(8.2 to 10.2)	9.1(8.1 to 10.1)	0.93	0.13±0.55
DA	10.3(9.7 to 10.9)	10.2(9.5 to 10.8)	0.84	0.15±0.54

Notes: * The mean and 95% confidence interval were provided. **LSVC was filled with thrombus in one patient, so LSVC was evaluated in nineteen patients.

**Figure 4:**
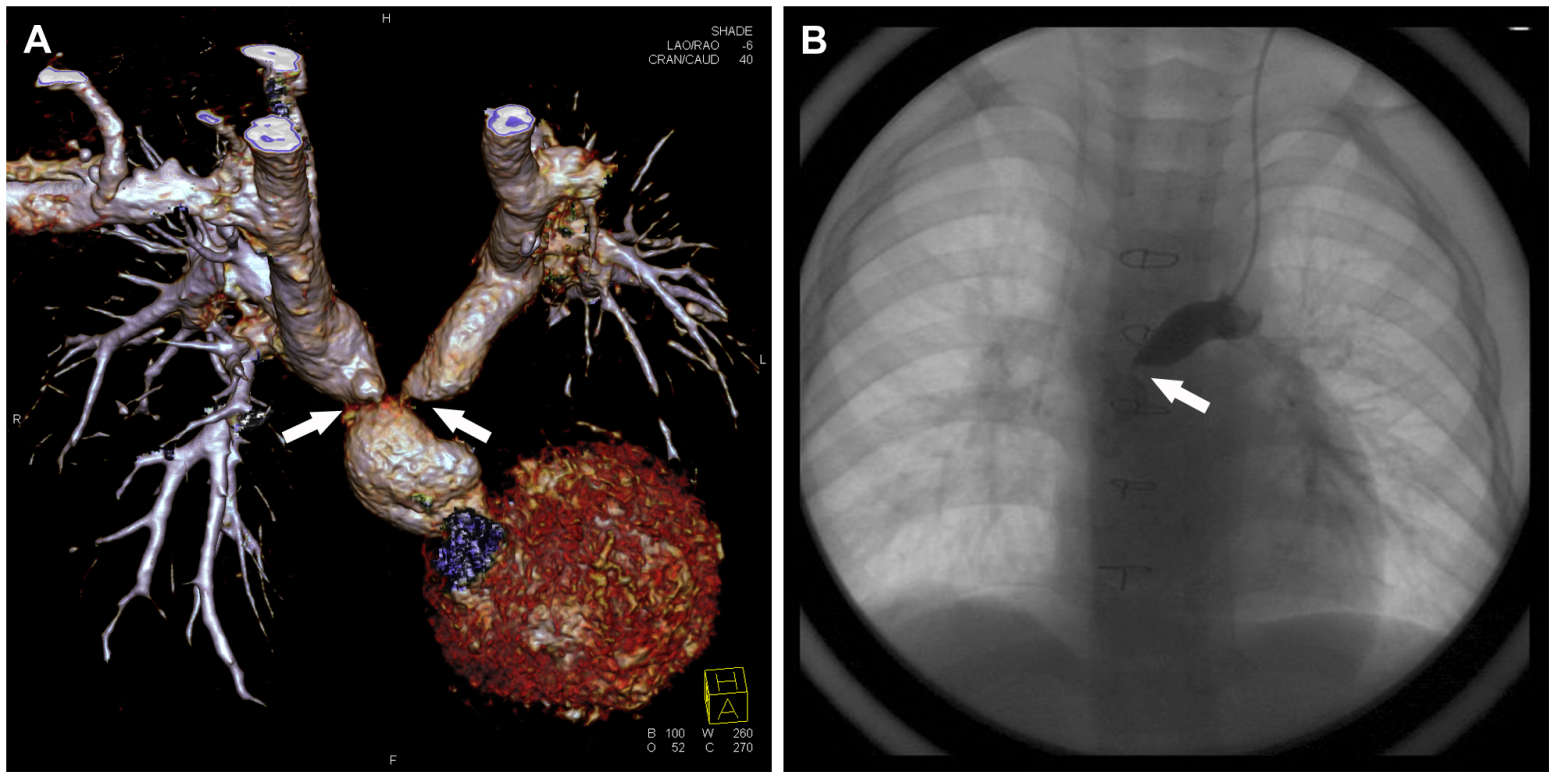
A 4.2-year-old boy underwent low-dose PGA scanning with 80 kV and 112 mAs/rot (effective dose, 0.46 mSv). **A**. Volume-rendered image shows severe stenosis (white arrow) in the root of bilateral PA. **B**. CCA image confirms the stenosis (white arrow) of LPA.

The comparison between SVC_max_ and SVC_0_ was as follows: ≤10%, n = 14; 10%–20%, n = 11; ≥20%, n = 14. The dilation was observed in 14 SVC by both CTA and CCA, and the corresponding SVC_max_ was 20% larger than SVC_0_ without exception. Therefore, we set ≥20% as the standard of dilation, and SVC_1_ was 20% larger than SVC_0_ in 10 of 14 dilated SVC. As for PA, 16 stenosis was found by CT and confirmed by CCA. There were 3 stenosis at the root (Fig. 4), 7 stenosis in the proximal part and 6 stenosis in the distal part of PA.

#### 3.2.2 The anastomotic relationship between SVC and PA

There were 24 SVC above PA and 15 SVC beside PA found by both CT and CCA. Considering dilation of SVC, we observed that 50% (12/24) SVC above PA dilated while only 13.3% (2/15) SVC beside PA dilated.

#### 3.2.3 Thrombosis, APCs and pulmonary AVMs

**Figure 5:**
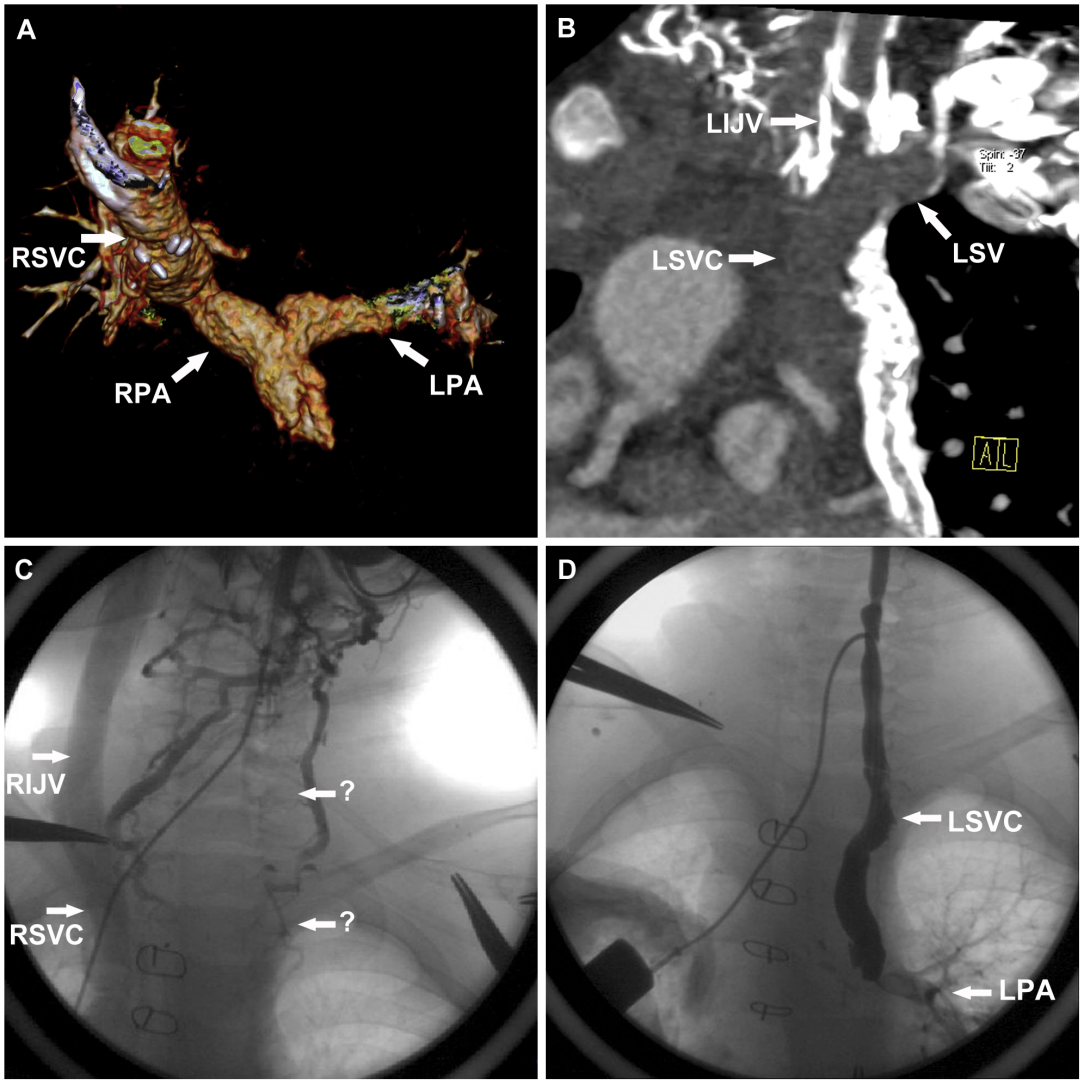
A 5-year-old boy underwent low-dose PGA scanning with 80 kV and 118 mAs/rot (effective dose, 0.48 mSv). **A**. Volume-rendered image shows the absence of LSVC. **B**. Multiplanar reformation image shows that thrombus was in the proximal part of left internal jugular vein, left subclavian vein and the whole course of LSVC. **C**. CCA image confirms the thrombus. **D**. CCA image shows the thrombus has been removed. *RSVC* right superior vena cava, *RPA* right pulmonary artery, *LPA* left pulmonary artery, *LSVC* left superior vena cava, *LIJV* left internal jugular vein, *RIJV* right internal jugular vein.

**Figure 6:**
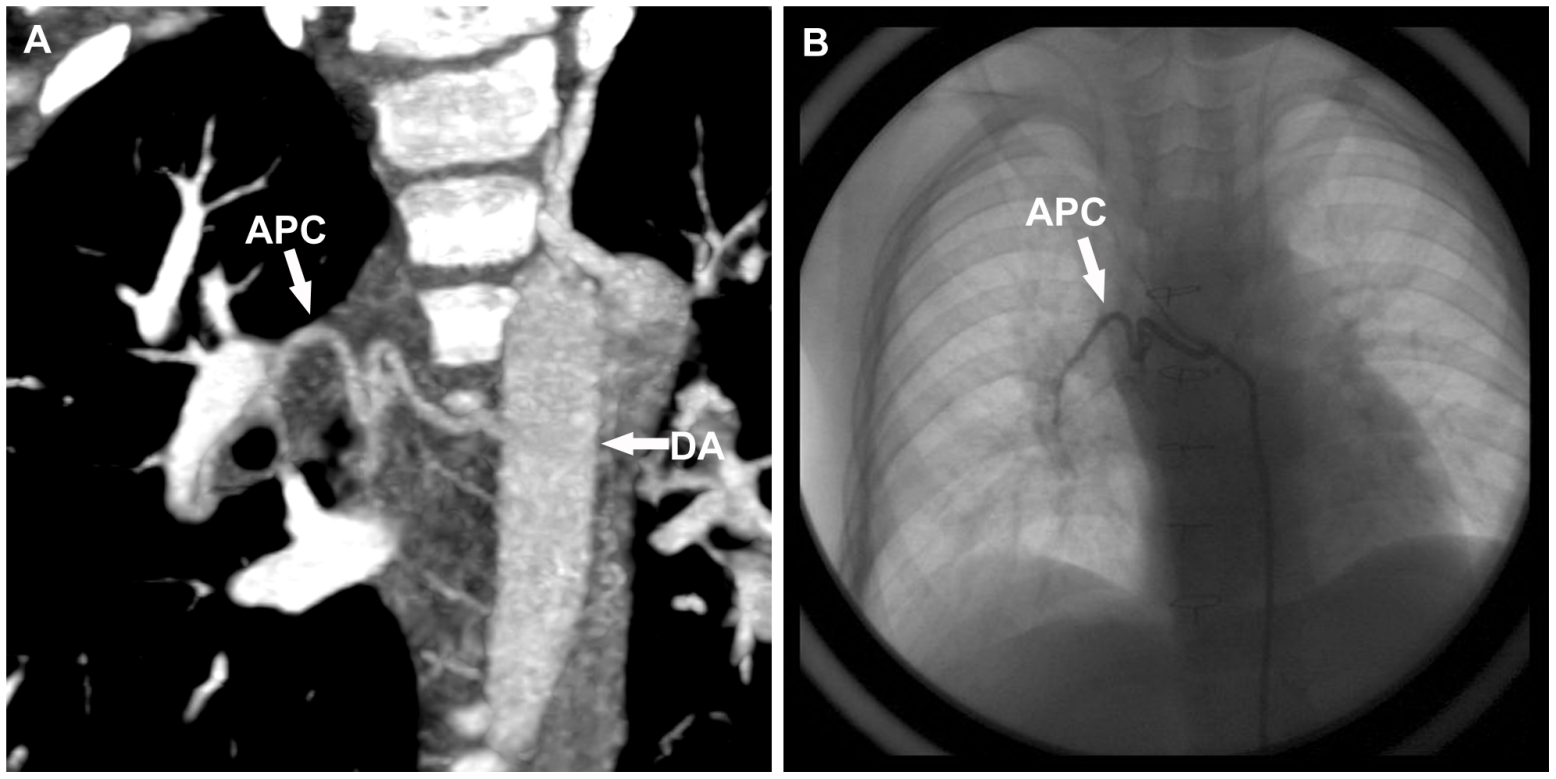
The same boy with Fig 4. **A**. maximum intensity projection image shows the existence of the collateral vessel. **B**. CCA image confirms the collateral vessel. *APC* aortopulmonary collateral vessel, *DA* descending aorta.

Thrombosis was found in 1 patient, locating in the whole course of LSVC as well as the proximal part of left internal jugular vein and left sub-clavian vein (Fig. 5A–C). The thrombus was removed by means of CCA a day later (Fig. 5D). A total of 33 APCs were identified by both CT and CCA among 13 patients (2 in 6 cases, 3 in 7 cases), and no APCs were identified in the remaining 7 patients (Fig. 6). The mean diameter of APC_max_ measured by CTA and CCA was 2.7±0.5 mm (2.1–3.6) and 2.5±0.4 mm (1.9–3.3) respectively, and there was a strong correlation (R^2^ = 0.83, *P*<0.001). Bland–Altman analysis demonstrated a systematic overestimation of the measurements by CTA (bias 0.11±0.20 mm).There were no pulmonary AVMs in 20 patients.

### 3.3. Radiation dose estimates

The mean volume CT dose index was 1.08±0.25 mGy (0.77–1.82), and the mean DLP was 12.1±4.1 mGy•cm (6.6–21.6), resulting in a mean estimated effective dose of 0.50±0.17 mSv (0.27–0.89). The mean dose-area product of CCA was 11.82±3.27 Gy•cm^2^ (7.49–16.39), resulting in an average effective dose of 4.85 ±1.34 mSv (3.07–6.72).

## Discussion

### 4.1 The challenge of CT scanning

The patients after bBDG had bilateral SVC. Then it comes to a question whether bilateral cavopulmonary anastomoses can be shown at the same time when the iodinated contrast medium was injected via unilateral peripheral veins. As we know, the blood containing the iodinated contrast medium mainly flushes into the unilateral PA from ipsilateral SVC after the injection. If the scanning had been performed at this time, higher attenuation would have been achieved at the ipsilateral SVC and PA of the injection while the contralateral SVC and PA would have lower attenuation. Therefore the scanning was delayed until the contrast medium came to the arteries of neck and upper limb and refluxed through the veins. Then the bilateral SVC was expected to achieve the preferable attenuation.

### 4.2 Postoperative morphological evaluation

**Figure 7:**
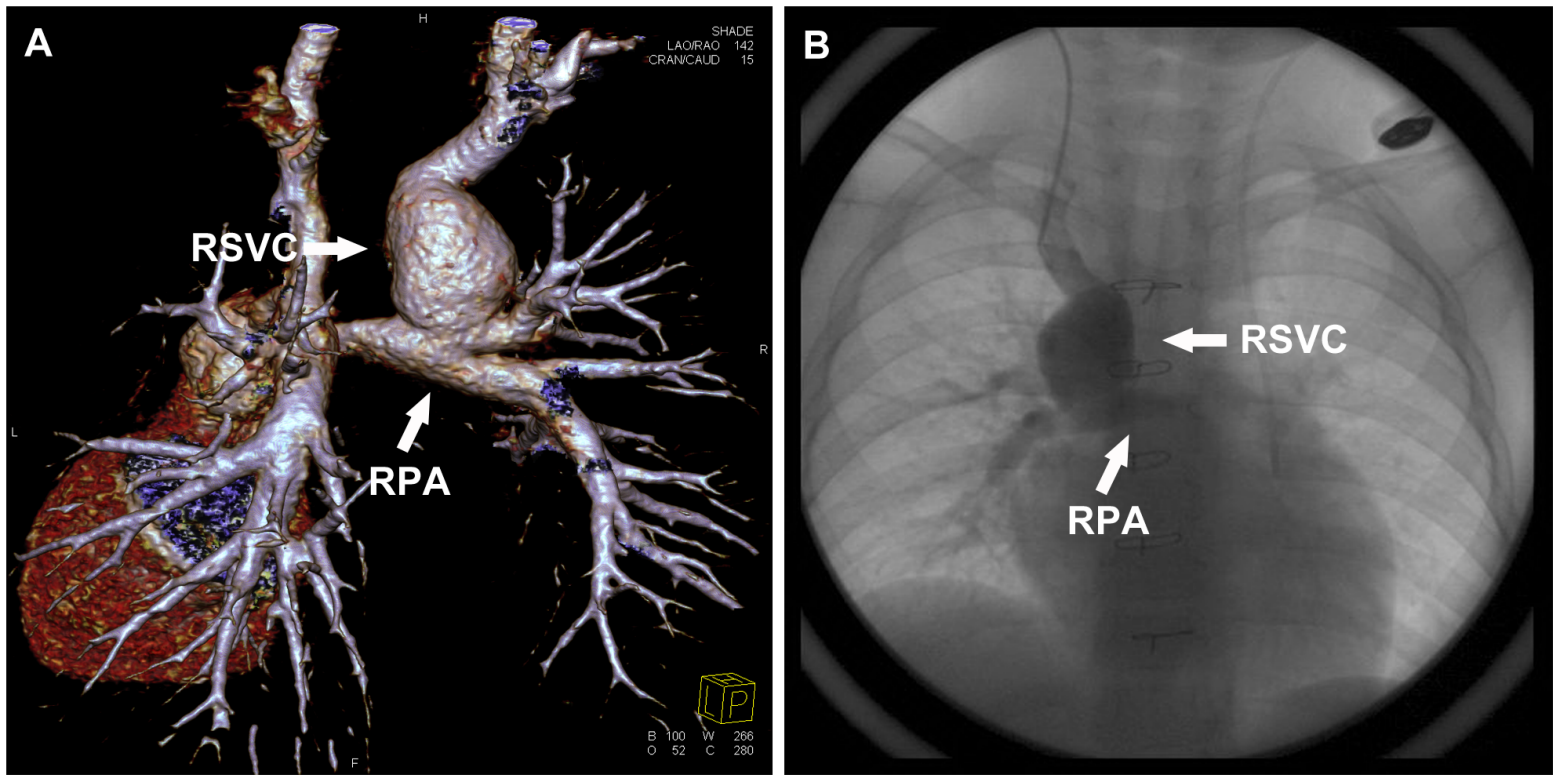
A 3.4-year-old boy underwent low-dose PGA scanning with 80 kV and 106 mAs/rot (effective dose, 0.44 mSv). **A**. Volume-rendered image (posterior view) shows the dilation of RSVC develops into aneurysm. **B**. CCA image (anterior view) confirms the aneurysm. *RSVC* right superior vena cava, *RPA* right pulmonary artery.

As the dilation of SVC implied the obstructed blood reflow or the unfavorable hemodynamics and the dilation sometimes developed into aneurysm (Fig. 7), much attention was paid to the measurement of SVC, and we found that the diameter of the proximal part of SVC was 20% larger than that of the distal part of SVC in 71.4% (10/14) dilated SVC. Therefore, the expansion of the proximal part of SVC reflected the dilation of SVC to some extent, and the proximal part of SVC should be detected by echocardiography in the follow-up in the event that the whole course of SVC is unable to be shown. With regard to PA, the stenosis of PA after pulsatile bBDG always draws the attention of pediatric cardiologists, and relief of stenosis by stent implantation is effective and can be realized safely to optimize pulmonary hemodynamics [30]. In our study, between the root and the anastomotic stoma of PA located ten stenosis (3 at the root, 7 in the proximal part), which may result from the surgery and made against the additional pulsatile pulmonary blood flow into the ipsilateral PA. The other 6 stenosis in the distal part of PA implied the pulmonary dysplasia.

To the best of our knowledge, there was no study discussing the anastomotic relationship between SVC and PA. We found that 50% SVC above PA dilated while only 13.3% SVC beside PA dilated. This phenomenon implied that SVC beside PA was less likely to dilate and the surgery may be optimized by anastomosing SVC beside PA. Thrombosis was one of the most severe complications in the patients after pulsatile bBDG [23], [24]. CTA was able to display the thrombus and surrounding tissues so as to provide sufficient information for thrombectomy. APCs are common after BDG and Fontan procedures [25]. John K, et al reported that the presence of APCs was diagnosed in at least one postoperative catheterization in 36% patients (71/196) [25]. Our study found that 65% patients (13/20) had collateral vessels. The proportion was much higher than 36%, which showed the prevalence of APCs after bBDG.

### 4.3 Different Imaging modalites

Our study demonstrated a close correlation between quantitative measurements by low-dose PGA scanning and CCA, although a small systematic overestimation by low-dose PGA scanning was observed. This overestimation may be attributed to partial volume effects and motion artifact by CT. The dilation of SVC, stenosis of PA, the anastomotic location between SVC and PA as well as thrombosis and APCs were also observed by both modalities. However, compared with the two-dimension CCA images, it was more accessible for doctors to understand the morphological changes by means of three-dimension reformation of CT images.

On the other hand, the dose of contrast medium for low-dose PGA scanning (2 ml/kg) was less than half of that for CCA (4.6 ml/kg), and the radiation dosage of low-dose PGA scanning (0.50 mSv) was much lower than that of CCA (4.85 mSv). Besides the potential risk of a large amount of contrast and radiation exposure [6], [9], other disadvantages of CCA include patient discomfort, bad availability, costs of hospital stay and a small but non-negligible risk of complications such as stroke, vessel dissection and pseudoaneurysm formation [6]. Therefore, except for the hemodynamic assessment, the diagnostic role CCA has largely been replaced by CT and other noninvasive imaging modalities.

Computed tomography scanners are widely available and easy to use in practice, and require shorter image acquisition time. With improvements in multi-detector technology, the spatial and temporal resolution of CT angiography has been dramatically improved to allow for imaging of even the most complex forms of CHD [16]–[21]. Up to now, there are four scanning modes for children with CHD, non-electrocardiogram-gated scanning, retrospectively electrocardiogram-gated technique, prospectively electrocardiogram-gated axial technique, and prospectively electrocardiogram-gated high-pitch spiral mode (HP mode). Non-electrocardiogram-gated imaging is usually used for the evaluation of extracardiac structural abnormalities [31], [32]. Retrospectively electrocardiogram-gated CT angiography was superior over non-electrocardiogram-gated acquisition when morphological evaluation of rapidly moving intracardiac or paracardiac structures, including the ascending aorta, cardiac valves and coronary artery, was required [33], [34]. However, high radiation due to its low pitch and overlapping data acquisition is still the major inherent limitation, especially for children [16], [35]. PGA technique, which has been widely used in children with CHD in recent years, usually provides adequate thoracic and coronary artery image quality in neonates, infants and young children with a low effective radiation [19]–[21], so we chose PGA technique as the evaluative method for patients after pulsatile bBDG. HP mode with a pitch of 3.4 provides high diagnostic accuracy and markedly reduces radiation dose, although image quality is mildly reduced [36]. Further studies may be conducted by means of HP mode if possible.

Echocardiography remains a first-line noninvasive imaging tool for evaluation of postoperative anatomy and ventricular function. However, echocardiography is operator-dependent and limited by the acoustic window, especially the post-surgical case with sternal wires and mediastinal scar tissue [8], [15]. Cardiac MRI has also been incorporated into the postoperative evaluation of patients after bBDG over the past few years due to its rapid development. Recent studies have demonstrated the ability of various CMR techniques to accurately evaluate congenital cardiac valve morphology and extracardiac thoracic vessels [37]–[39]. As for flow measurement, phase-contrast MRI can quantify aorto-pulmonary collateral flow after bidirectional cavopulmonary connections [40] and a negative correlation was found between aorto-pulmonary collateral flow and the pulmonary artery index [41]. However, metallic implanted devices, such as stents and embolization coils may be associated with susceptibility artifacts, which can cause substantial limitation for MRI examinations. Furthermore, CMR is resource-intensive, technically challenging, and requires general anaesthesia or deep sedation for most children to avoid movement and imaging distortion [10]–[12]. Anesthetics by gas or by continuous infusion must be maintained throughout the MRI study [13]. Therefore, the operational risks of general anesthesia or deep sedation must be considered for these patients during the long acquisition time of CMR [14].

### 4.4 Limitation

Our study has several limitations. First of all, we included a relatively small group of patients as the number of the children repaired via pulsatile bBDG was relatively small. What's more, radiation is the inherent limitation of CT, and the HP mode of 128-slice dual-source CT may further lower the radiation dose.

## Conclusion

CT angiography with a low-dose PGA scanning is an accurate and reliable noninvasive examination in the assessment of morphological changes in patients with pulsatile bBDG.
